# Acceptability and Preliminary Evaluation of a Campus-Integrated Digital Platform (Fruto) for University Students’ Mental Health Help-Seeking: Sequential Mixed Methods Study

**DOI:** 10.2196/78930

**Published:** 2026-06-22

**Authors:** Myungsung Kim, Hyorim Kim, Jeong-in Heo, Seonmi Lee, Orane Farrah Lahcine, Sangil Lee, Hwang Kim, Dooyoung Jung

**Affiliations:** 1 Graduate School of Health Science and Technology Ulsan National Institute of Science and Technology Ulsan Republic of Korea; 2 School of Digital Humanities and Computational Social Sciences Korea Advanced Institute of Science and Technology Daejeon Republic of Korea; 3 School of Liberal Arts Ulsan National Institute of Science and Technology Ulsan Republic of Korea; 4 Department of Design Ulsan National Institute of Science and Technology Ulsan Republic of Korea; 5 Mind Care & Growth Center Korea Advanced Institute of Science and Technology Daejeon Republic of Korea

**Keywords:** app usability, digital mental health, help-seeking, mixed methods, mobile health, multidomain platform, stigma reduction, university students

## Abstract

**Background:**

College students are at heightened risk for mental health problems; yet, professional help-seeking remains low. Although digital mental health tools can improve accessibility and reduce stigma, many focus on isolated functions, such as self-screening, psychoeducation, or symptom management, and are not fully integrated with campus counseling services. Multidomain platforms that combine screening, mental health information, counseling access, and campus service navigation may support help-seeking in university settings; however, their acceptability and implementation value remain underexplored.

**Objective:**

This mixed methods study examined the refinement and preliminary evaluation of Fruto, a campus-integrated, multidomain app developed to support university students’ help-seeking attitudes and counseling-related beliefs in a real-world counseling-center setting. Phase 1 used scenario-based prototype sessions to analyze students’ interactions with the platform and inform refinement. Phase 2 assessed whether 8 weeks of Fruto use was associated with pre-post changes in attitudinal and counseling-related outcomes.

**Methods:**

We conducted a 2-phase, mixed methods study. Phase 1 involved vignette-based prototype sessions and semistructured interviews with 16 students to explore user experiences with an early version of Fruto. Scenario-based tasks facilitated feedback on the platform, and thematic analysis identified design implications that guided refinement. Phase 2 involved an 8-week single-group pre-post evaluation. A total of 109 students completed the baseline survey, and 70 provided follow-up responses. Surveys assessed help-seeking attitudes, counseling-related beliefs, and perceived app quality. Linear mixed effects models examined pre-post changes using all available data, and exploratory baseline-adjusted regressions examined the association between overall perceived app quality and postuse outcomes.

**Results:**

Scenario-based prototype sessions elicited actionable feedback on how students might use Fruto in realistic help-seeking contexts. Qualitative findings identified 3 refinement priorities that informed subsequent app updates: trusted and identifiable content providers, seamless integration across app features, and relatable self-discovery content to lower psychological barriers to app use. Following these refinements, Phase 2 assessed 8-week pre-post changes using linear mixed effects models. Fruto use was associated with significant increases in positive help-seeking attitudes (B=0.884, SE 0.284, 95% CI 0.327-1.441; *P*=.002) and positive counseling expectations (B=1.585, SE 0.541, 95% CI 0.526-2.645; *P*=.003). No significant changes were observed in negative attitudes, negative counseling beliefs, or socially supportive beliefs. In exploratory baseline-adjusted regressions, overall perceived app quality was associated with positive counseling expectations, but not with positive help-seeking attitudes.

**Conclusions:**

Fruto shows promise as a campus-integrated, multidomain platform associated with more favorable help-seeking attitudes and counseling expectations among university students. These findings suggest that multidomain platforms may strengthen positive, approach-oriented beliefs toward professional support. Future studies with longer follow-up and objective usage or service-use data are needed to examine whether attitudinal changes translate into help-seeking behavior.

**Trial Registration:**

Clinical Research Information Service KCT0010622; https://cris.nih.go.kr/cris/search/detailSearch.do?seq=30274&search_page=L

## Introduction

### Mental Health Challenges and Help-Seeking Behavior Among University Students

During the transition from adolescence to adulthood, university students face new roles and environments while managing social, economic, and academic pressures. This life stage, often termed “emerging adulthood” [1], is also when most lifetime mental illnesses first manifest, making it a critical developmental period with substantial individual and societal consequences [2]. Mental health problems among university students are therefore well-documented and pose a significant threat to well-being. In the United States, over one-third of students report depressive symptoms that impair their daily functioning, and approximately 10% seriously consider suicide each year [3]. Yet, despite growing recognition of this burden, the implementation of accessible, acceptable, and student-centered support systems remains limited, particularly regarding how universities can encourage timely professional help-seeking before students’ difficulties become severe.

These concerns are especially relevant in South Korea, where university student mental health must be understood within the country’s specific context of high psychological distress, academic pressure, and limited use of professional mental health services [4]. South Korea continues to report suicide rates above the Organisation for Economic Co-operation and Development average, and Korean university students report substantial distress related to interpersonal relationships, employment, and career decisions, with prior Korean studies documenting considerable employment- and career-related distress, including job-seeking stress, depressive symptoms, and job-search anxiety [5-7]. According to the Ministry of Health and Welfare, only 22.2% of Koreans seek professional mental health services, compared with 41.3% in the United States and 46.5% in Canada [8]. This treatment gap is concerning because delayed help-seeking may allow student distress to worsen before they reach campus counseling or professional support.

Low use of professional mental health services among students does not necessarily reflect a lack of interest in support. Prior evidence suggests a gap between students’ help-seeking intentions and their actual use of professional services, indicating that many students may recognize a need for help yet still face barriers to accessing care. These barriers are multifaceted and include a preference to manage problems independently, lack of time, limited knowledge of available resources, and concerns about privacy or stigma. In Asian cultural contexts, where maintaining social reputation is highly valued, students may also be reluctant to disclose mental health concerns [9-11]. These cultural and practical barriers can reinforce self-stigma, delay professional help-seeking, and reduce students’ likelihood of accessing support before difficulties worsen [12,13].

### Support Programs and Online Interventions

As awareness of student mental health concerns grows, universities worldwide are facing increasing demand for mental health services [14]. On-campus counseling centers play a key role in identifying and addressing students’ emotional challenges, and face-to-face counseling has been shown to reduce symptoms of depression, anxiety, hostility, academic distress, and social anxiety [15]. Universities are increasingly adopting stepped-care models that match students to different levels of support according to need, integrating peer support, group therapy, and psychiatric care alongside traditional face-to-face counseling [16]. This approach can extend support beyond individual counseling by offering earlier, more accessible, and less resource-intensive options at different points in students’ help-seeking needs.

Within this stepped-care approach, digital mental health interventions can help expand support by offering students accessible, low-intensity, and less stigmatizing support options [17]. University students are often comfortable using digital platforms and may value the anonymity and flexibility they provide, making online support particularly appealing [12]. Moreover, beyond symptom-focused digital interventions, help-seeking-oriented programs have aimed to improve mental health literacy and increase students’ willingness to seek professional support [18]. These approaches often use psychoeducation or self-screening to help students recognize mental health concerns and understand the value of support [19,20]. Still, awareness alone may be insufficient if students do not know how to interpret screening results or what concrete steps to take afterward. Self-screening tools, in particular, may lead to confusion or inaction when they are not paired with clear follow-up guidance.

Yet many existing digital programs focus on single clinical targets, such as depression, anxiety, or general well-being, and are frequently delivered as stand-alone self-help or mobile-based cognitive behavioral therapy [12]. In addition, when digital tools are not aligned with the counseling approaches and service structures already used on campus, a mismatch may emerge between app-based support and offline care pathways [21]. Although these tools can be useful, they may not fully address students’ broader help-seeking needs, such as understanding available services, interpreting self-screening results, reducing hesitation, and taking concrete steps toward campus-based support. Therefore, digital platforms designed for university settings should not only provide information or screening, but also connect students to appropriate next steps, including campus counseling and other in-person support services [21].

### Study Purpose

Taken together, these gaps suggest a need for campus-integrated digital platforms that do more than provide isolated information or symptom-focused self-help. In response, this study examines the real-world acceptability, user-informed refinement, and preliminary evaluation of Fruto, a campus-integrated, multidomain platform developed to support university students’ mental health help-seeking attitudes and counseling-related beliefs. Fruto incorporates elements commonly used in digital mental health tools, such as psychoeducation and self-screening, but embeds them within a unified help-seeking ecosystem linked to university counseling services.

In the initial stage of the study, we conducted vignette-based semistructured interviews followed by thematic analysis to examine how students perceived and interacted with an early version of Fruto. This phase addressed 2 research questions (RQs): (RQ1) how university students with varying usage motivations engage with and perceive multidomain help-seeking platforms, and (RQ2) what design principles are essential for ensuring that such a platform functions effectively within a real-world campus environment.

Based on the initial findings, we refined Fruto and conducted a pre-post evaluation among campus student users to examine whether platform use was associated with changes in help-seeking attitudes and counseling-related beliefs. This second phase addressed one research question: (RQ3) whether the use of a multidomain help-seeking platform is associated with changes in university students’ help-seeking attitudes and counseling-related beliefs. To address this quantitative research question, we established two hypotheses: (H1) students who use Fruto for 8 weeks will show improved attitudes toward seeking professional help, and (H2) students who use Fruto for 8 weeks will demonstrate more positive perceptions of counseling.

In summary, this study first used scenario-based qualitative methods to examine how students engage with a theoretically grounded, multidomain help-seeking platform (RQ1) and to identify design implications for real-world campus implementation (RQ2). These findings informed the refinement of Fruto before a campus-wide pre-post evaluation. The study then examined whether 8 weeks of Fruto use was associated with changes in help-seeking attitudes and counseling-related beliefs (RQ3). This integrated approach aims to clarify how multidomain digital platforms can support help-seeking attitudes and counseling-related beliefs in university settings.

## Methods

### Multidomain Platform: Fruto

**Figure 1:**
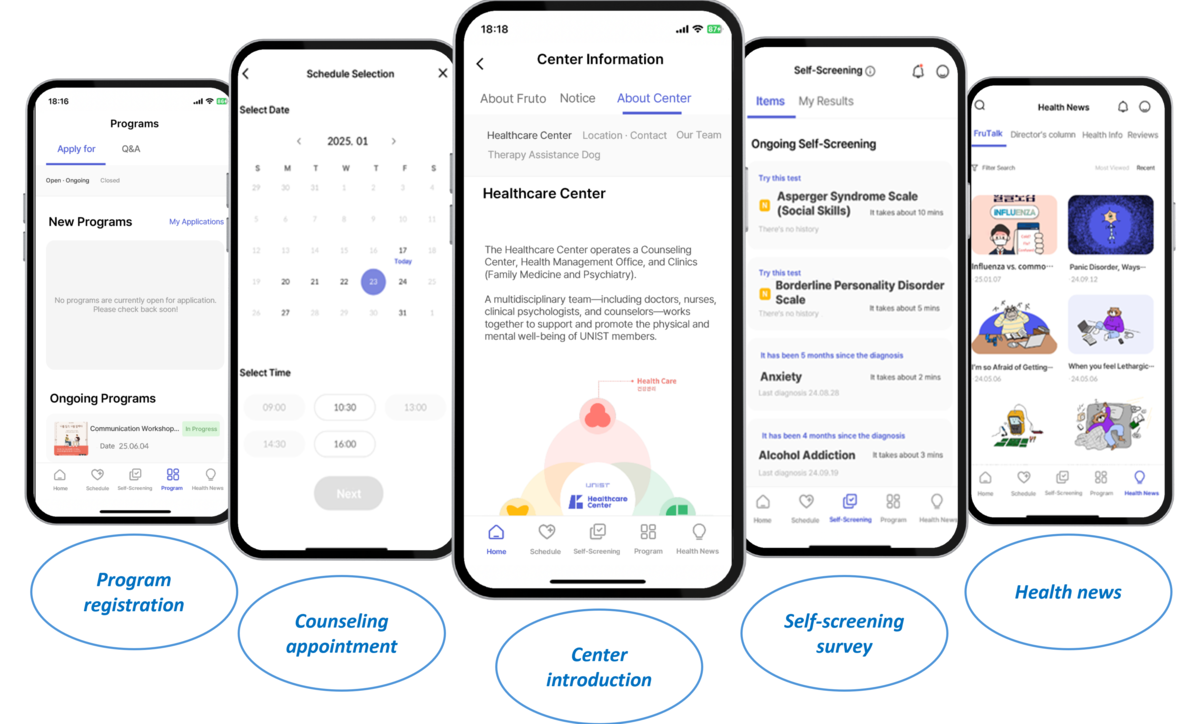
Main user interface components of Fruto.

We conceptualize Fruto as a multidomain help-seeking platform, distinct from traditional multicomponent apps that deliver therapeutic tools or multimodule apps that provide sequential intervention content. Each feature within Fruto was designed to support students across different problem and functional domains, thereby facilitating multiple pathways toward mental health help-seeking. The platform includes 5 core features, each developed in-house by the research team and integrated into a cohesive user interface (UI). The home screen includes a navigation bar linking to each core function, along with key announcements and informational resources. The platform design was informed by existing literature on help-seeking behavior and grounded in relevant theoretical frameworks. Figure 1 presents the main UI components. Full UI screenshots and feature descriptions are provided in Multimedia Appendix 1.

The five core components are described below:

1. Self-screening survey: self-screening is commonly used in mobile mental health interventions to help users reflect on their symptoms and has been recognized as potentially enhancing motivation for self-care or professional support [22]. Previous research has emphasized limitations when self-screening is offered in isolation, as it may leave users uncertain about subsequent steps [23] and does not necessarily lead to direct improvement in depressive symptoms [24]. When results are presented clearly and in a user-friendly manner, without requiring clinical interpretation, they can also assist professionals in making informed treatment decisions [24]. Fruto offers mobile-optimized versions of validated screening tools, including the Patient Health Questionnaire-9 for depression, Generalized Anxiety Disorder-7 for anxiety, Alcohol Use Disorders Identification Test–Korean version for alcohol use, Adult ADHD Self-Report Scale Version 1.1 for attention difficulties, Pittsburgh Sleep Quality Index–Korean version for sleep quality, and the P4 Suicide Risk Screener for suicide risk. Upon completion, users receive immediate, color-coded feedback accompanied by an explanatory summary. To address the limitations of stand-alone screening, Fruto provides follow-up guidance, such as counseling referrals and tailored content recommendations for users with elevated scores or risk indicators. With user consent, these results are also shared with an affiliated counselor, who may incorporate them into consultations or platform-based interventions.
2. Health news: university students often prefer text-based resources, particularly in settings such as dormitories and classrooms where privacy is limited, over audio or visual materials [25]. This preference may be especially relevant in contexts where stigma around mental illness persists [26]. Fruto addresses these specific barriers by offering professionally curated, text-based content to enhance mental health literacy and reduce stigma [8,25]. Developed in collaboration with licensed counselors and psychiatrists, the articles cover a wide range of topics, including psychiatric disorders, well-being, mindfulness, and available campus services. To create a more personalized experience, the content is also tailored to users’ self-screening results.
3. Counseling appointment: while online interventions can help reduce psychological barriers to help-seeking [10,21], effective mental health care often requires integration with offline services. However, many students find scheduling appointments in person or over the phone burdensome, especially when experiencing depression-related apathy or social anxiety [27]. Previous studies have shown that digital tools, such as online booking systems, can help mitigate these challenges [28]. Fruto enables students to schedule on-campus counseling sessions directly through the mobile application. The system includes a brief intake form that gathers information about symptoms and concerns, encouraging self-reflection before the session and helping counselors prepare for more efficient, informed consultations.
4. Program registration: although campus mental health services can offer scalable, tailored support and help reduce stigma across the broader student population via wellness programs (eg, group therapy, mindfulness training, and personality assessments) [16,29], participation rates remain low due to poor publicity and limited access to information [30]. Integrating these programs into digital platforms has been proposed as a way to enhance accessibility and sustainability [25,31]. Fruto provides a dedicated interface for wellness program registration, allowing counselors to share detailed information about each program, its eligibility criteria, and its schedules. Students can easily browse ongoing, upcoming, and past sessions and register directly through the platform. An anonymous comment feature also allows students to express preferences and suggest new topics, helping practitioners better align future programs with student needs.
5. Center introduction: reliable information about on-campus services is essential for building trust and encouraging student engagement. Fruto provides basic information and emergency contact details for university mental health centers [32,33]. Important updates, such as operational hours and event announcements, are regularly posted via a dedicated bulletin board to keep students informed and connected.

To make the platform’s design logic more explicit, we mapped each Fruto feature to theory-informed mechanisms relevant to help-seeking, drawing on the theory of reasoned action/theory of planned behavior tradition. Rather than assuming that all features influence help-seeking in the same way, we conceptualized self-screening, psychoeducational content, counseling access, program registration, and center information as targeting different but complementary pathways, including attitudes, perceived norms, and perceived behavioral control. Table 1 maps Fruto’s main features, functions, proposed mechanisms, and theoretical constructs.

**Table 1:** Mapping of Fruto features to theory-informed help-seeking mechanisms and constructs.

Fruto feature	Primary function	Theoretical construct	Proposed mechanism
Self-screening	Symptom recognition and self-monitoring	Attitude toward help-seeking	Makes psychological status more concrete and increases the perceived relevance of professional support
Health news	Mental health literacy and stigma reduction	Subjective norms + attitude	Normalizes help-seeking through expert-curated information and reduces stigma
Counseling appointment	Lowering access barriers	Perceived behavioral control	Simplifies action steps and increases confidence in accessing counseling
Program registration	Participation in preventive programs	Subjective norms	Encourages participation in campus well-being activities and normalizes support use
Center introduction	Service awareness and trust	Attitude + perceived behavioral control	Increases trust in campus services and clarifies how to use them

In a subsequent step, we contextualized Fruto within existing university-oriented digital mental health platforms by conducting a descriptive app comparison using an adaptation of the Lancet Digital Health app evaluation framework [34]. Fruto, TimelyCare [35], YOU at College [36], and IntelliCare for College Students [25] were reviewed based on publicly available information, and each criterion was rated on a 3-point scale through consensus discussion among the research team. The comparison was descriptive rather than evaluative and is provided in Appendix 2.

### Technical Implementation and Data Governance

Fruto was deployed within an Ulsan National Institute of Science and Technology (UNIST)-managed cloud environment. Data transmitted between the client and server was protected using Transport Layer Security in accordance with the university cloud network configuration, and individual identifiable information was encrypted at rest using AES-256. User authentication initially relied on email-and-password login and was later expanded to support single sign-on for university-affiliated users, while retaining email-based login for general users. Access to personal data was restricted according to service roles; in the evaluated versions, counselors could access only information voluntarily submitted or shared by students through the platform for service provision, and administrative access to personal information was logged and controlled. Explicit consent procedures were incorporated into both account registration and counseling request workflows.

### Study Design

**Figure 2:**
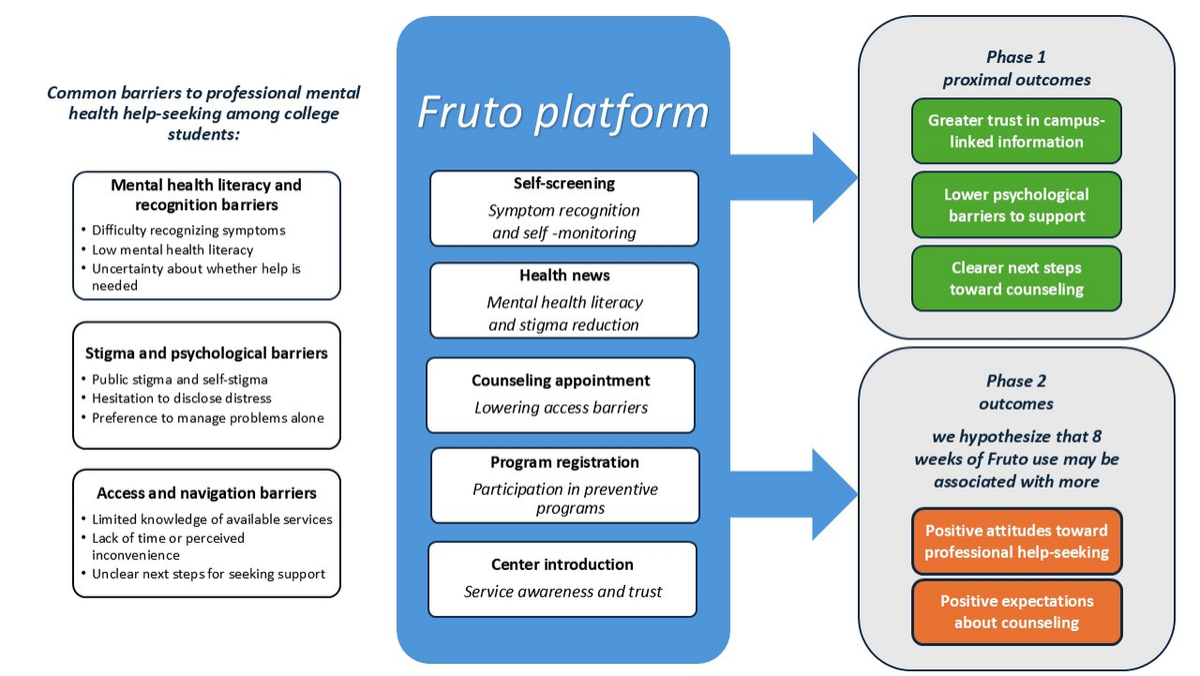
Joint display linking Phase 1 themes, corresponding design refinements, and related domains assessed in Phase 2.

We conducted a sequential mixed methods study to explore the initial acceptability of Fruto’s design and features, followed by an evaluation of its potential contribution in a real-world university setting. In Phase 1, we qualitatively investigated how a group of 16 undergraduate and graduate students experienced the prototype. Insights from this phase guided revisions to the platform’s design and functionality. In Phase 2, we conducted a large-scale survey to assess changes in help-seeking attitudes and counseling-related beliefs after the platform’s implementation. Figure 2 presents a joint display linking Phase 1 themes, corresponding design refinements, and related domains assessed in Phase 2.

### Participant Recruitment

#### Phase 1: Qualitative Study

Since Fruto was designed for the general university student population, all enrolled undergraduate and graduate students were eligible to participate. Recruitment was conducted through Every Time, a Korean online campus community, where a call for beta testers was posted. Participants provided informed consent and received 20,000 KRW (approximately US $13.6) as compensation for their participation.

#### Phase 2: Quantitative Study

After incorporating improvements based on Phase 1 findings, Fruto was distributed campus-wide, and recruitment for Phase 2 began. A new survey invitation was posted and promoted through informational posters across campus. Participants were instructed to complete pre- and postuse surveys and to engage freely with the app over an 8-week period. Upon completion, they received compensation of 10,000 KRW (approximately US $6.8).

### Ethical Considerations

Phase 1 consisted of vignette-based prototype sessions and interviews focused on user experience. After completion of Phase 1, the protocol and study materials were submitted to the UNIST Institutional Review Board, which determined that the activity met the criteria for exemption from full review (approval number UNISTIRB-26-020-C). Participants provided informed consent for participation and audio recording, and interview data were stored securely under institutional privacy procedures. In addition to research consent procedures, the platform incorporated explicit service-level consent steps for account registration and counseling requests. Personal information was handled according to institutional privacy and security procedures, including encrypted transmission and storage, controlled access, and access logging.

Phase 2, which involved the real-world deployment and evaluation of the Fruto platform, was approved by the Ulsan National Institute of Science and Technology Institutional Review Board (approval number UNISTIRB-24-047-A). Participants received detailed online information about the study purpose, procedures, and potential risks and provided informed consent electronically via Google Forms before participation. Participants were compensated for their time. Phase 1 participants received KRW 20,000 (US $13.6), and Phase 2 participants received KRW 10,000 (US $6.8) upon completion of the study procedures.

### Qualitative Data Collection and Analysis

#### Study Procedure

A vignette-based semistructured interview method was developed to present short scenarios featuring hypothetical individuals in specific situations, prompting participants to reflect on how they might respond in similar contexts [37]. We used this approach to reduce psychological burden and encourage honest feedback on sensitive or complex issues. Prior research suggests that vignette-based techniques allow individuals to express their attitudes, perceptions, and values from a third-person perspective [38].

We designed four vignettes reflecting distinct motivations for using mental health apps, based on prior research identifying key drivers among university students [39]. While many students are motivated by personal stress or needs, external influences such as peer encouragement or previous exposure to mental health issues may also influence app use [40-42]. The four vignette types were (1) supporting a peer in distress, (2) seeking help for oneself, (3) casual curiosity, and (4) maintaining well-being despite no current problems.

To capture a wider range of intended use contexts while keeping the prototype session manageable, each participant was presented with 2 vignettes in sequence and asked to role-play the main character while exploring the prototype version of Fruto. After a brief app overview, participants freely interacted with its features for approximately 10 minutes, during which their screen activity was recorded. All sessions were conducted face-to-face in a small office or laboratory setting by one of two researchers (MK or Hyorim K). To simulate natural usage, the researcher temporarily left the room during this time.

Following the interaction, a one-on-one semistructured interview lasting approximately 20 minutes was conducted. The interview focused on participants’ app usage, identifying helpful or problematic features, and gathering suggestions for improvements. The interview guide was developed in advance and used consistently across participants; no substantive modifications were made during data collection. The full interview guide is provided in Multimedia Appendix 3.

To ensure that the prototype was evaluated across different help-seeking contexts, the scenario design was structured around distinct usage motivations, as described below.

#### Scenario Design Based on Usage Motivation

Four user types were defined for vignette construction: peer supporter (PS), students who downloaded the app to support friends struggling with mental health issues; help seeker (HS), students whose mental health problems have significantly disrupted their daily lives and prompted them to seek support through the app; curious explorer (CE), students who discovered the app through campus promotions or incentives and tried it out of curiosity; and proactive maintainer (PM), students without current concerns who wished to proactively manage their mental well-being based on personal or vicarious experiences. The original vignette scripts provided to participants are included in Multimedia Appendix 4.

#### Data Management and Thematic Analysis

All interviews were audio-recorded with participants’ informed consent. The 2 interviewers (MK and Hyorim K) manually transcribed the recordings of the interviews they had conducted. Transcripts were securely stored in encrypted cloud storage, accessible only to the research team.

We conducted an inductive thematic analysis to identify meaningful patterns across participant responses [43,44]. This approach was appropriate for exploring how students experienced the prototype across different vignette contexts and for generating design-related insights. MK and Hyorim K repeatedly read all transcripts to familiarize themselves with the dataset and recorded initial analytic notes. Transcripts were then organized in Microsoft Excel into meaning units, with each unit tagged according to vignette type and app feature. The 2 researchers collaboratively assigned inductive codes to these meaning units and iteratively refined the coding framework by merging overlapping codes and clarifying ambiguous codes through repeated analytic discussions.

Related codes were then grouped into candidate themes. Theme development was guided not only by recurrence across interviews but also by the relevance of the patterned responses to the study questions concerning user experience and design implications. Candidate themes were reviewed and refined through repeated discussions between MK and Hyorim K, with additional feedback from coauthors with expertise in digital health, psychiatry, clinical psychology, and design. The first author subsequently refined the final thematic structure, finalized theme labels and descriptions, and selected representative quotations [44,45].

#### Sample Adequacy, Reflexivity, and Trustworthiness

A total of 16 students participated in Phase 1. Each participant completed 2 vignette scenarios; therefore, the final qualitative dataset comprised 32 vignette-based use episodes, with 8 cases for each vignette type. Rather than applying formal saturation as a fixed stopping rule, we considered sample adequacy in relation to the focused aims of this design-oriented qualitative phase and the coverage achieved across the four intended use contexts. Data collection concluded when all vignette conditions had been adequately represented, and subsequent interviews yielded largely repetitive, design-relevant insights [45-47].

Throughout the analytic process, the researcher's reflexivity was considered. The first author had been involved in the Fruto project since its early planning stages, had prior experience in digital mental health research and qualitative analysis, and had collaborated with the campus counseling center for several years. This position supported strong contextual familiarity with the platform and the institutional setting, but also required reflexive awareness of prior assumptions about the platform and campus mental health services. In contrast, the second author had joined the project more recently, came from a different university background, and brought a relatively less embedded perspective to the data. These differing positions were used during analytic discussions to question assumptions and refine interpretations [45,48].

To enhance trustworthiness, we used repeated engagement with the full dataset, illustrative quotations, multidisciplinary review of developing themes, and documented coding records. Credibility was supported through repeated discussion of interpretations and direct linkage between themes and participant accounts; dependability through use of a consistent interview guide and documented analytic procedures; confirmability through critical review by coauthors from different disciplinary backgrounds; and transferability through contextual description of the institutional setting and platform characteristics [44,48,49].

#### Design Iteration Process

After completion of the Phase 1 interviews, the research team synthesized the main user experience issues and design implications and organized them into actionable refinement priorities. These priorities were reviewed together with the UI designer and campus counseling center staff to ensure that the proposed changes were both user-centered and feasible within the counseling service workflow. Based on this discussion, the team translated the findings into concrete interface, content, and service-flow revisions before Phase 2. The UI designer incorporated these agreed-upon revisions into updated interface plans, while counseling center staff provided input on how the revised features should support service delivery and student guidance. The updated plans were then communicated to the development team for implementation, followed by several iterative reviews before campus-wide launch [25,32,50].

### Quantitative Data Collection and Analysis

#### Study Procedure

The second phase used a single-group pre-post design. Following Fruto’s official launch in September 2024, students who completed the preuse survey were invited to freely use the app for 8 weeks. At the end of this period, follow-up surveys were emailed to participants. For the primary repeated-measures analyses, we used all available outcome data from the 109 participants who completed the baseline survey and the 70 participants who provided postuse data.

The quantitative phase was not designed to isolate the effects of individual Fruto components or to test feature-specific behavioral mechanisms. Its purpose was instead to examine whether the integrated multidomain platform, used as a whole under real-world campus conditions, was associated with favorable attitudinal and perceptual outcomes during this formative stage of evaluation.

#### Sample Size Estimation

A priori power analysis was conducted using G*Power (Universität Düsseldorf) to estimate the sample size required for a single-group pre-post design. Prior digital interventions targeting mental health literacy and help-seeking intentions have reported small-to-medium effects. A meta-analysis of digital interventions aimed at improving mental health literacy reported an effect size of approximately 0.45, with similar estimates for stigma reduction of approximately 0.33 [51]. Studies targeting help-seeking intentions have reported effect sizes around 0.48, although these effects were not always statistically significant [52]. Given the preliminary, real-world nature of this study and the use of voluntary campus users rather than a clinical population, we considered several planning scenarios. A conservative scenario using an expected effect size of *d*_z_=0.30 indicated a required sample size of approximately 89 participants with α=.05 and power=.80. Small-to-medium scenarios required smaller samples, including approximately 75 participants for *d*_z_=0.33, 67 participants for *d*_z_=0.35, and 52 participants for *d*_z_=0.40. To account for potential attrition over the 8-week period, we aimed to recruit more than 100 students for the preuse survey. Although the baseline recruitment target was met, the number of participants who completed the postuse survey was below the most conservative complete-case target but within the range required for detecting small-to-medium pre-post effects. Therefore, Phase 2 findings should be interpreted as preliminary.

#### Measures

In both pre- and postintervention surveys, participants reported their attitudes toward professional help-seeking and perceptions of counseling. App usability and perceived quality were assessed at postintervention.

Attitudes Toward Seeking Professional Psychological Help was used to measure students’ trust in and perceived need for counseling services. It includes 2 subscales, positive and negative attitudes, and has been validated for use with Korean populations [53,54].

The Beliefs and Expectations About Counseling Scale was used to assess students’ beliefs about counseling. Grounded in multiattribute attitude theory and the theory of reasoned action, this scale assesses students’ beliefs about counseling. Validated among both US and Korean university students, it includes subdomains such as positive expectations, negative endogenous beliefs, and socially supportive beliefs [55,56].

The user version of the Mobile App Rating Scale (uMARS) was used after app use to assess app quality from a general user perspective. It includes subscales for engagement, functionality, aesthetics, information, and subjective quality [57]. As no official Korean version was available, the research team used a Korean translation of the uMARS for exploratory purposes in this study. The translated items were reviewed by bilingual members of the research team for conceptual clarity, although formal back-translation and cross-cultural validation were not performed. The Korean translation of the uMARS showed acceptable internal consistency in this sample (Cronbach α=0.77 for engagement, 0.79 for functionality, 0.80 for aesthetics, 0.79 for information, and 0.72 for subjective quality), but it was used for exploratory purposes only and should not be interpreted as a formally validated Korean version.

#### Data Collection, Pre-Post Analysis, and Sensitivity Analysis for Missing Follow-Up Data

Survey data were collected using Google Forms, with all participants providing informed consent for research use before completing the survey. Internal consistency was evaluated using Cronbach α for each construct. Composite reliability (CR) and average variance extracted (AVE) were also calculated from baseline responses to examine convergent validity. CR values of ≥0.70 and AVE values of ≥0.50 were interpreted as indicating acceptable convergent validity [58]. Potential attrition bias was examined by comparing participants who completed only the baseline survey with those who also provided postintervention data. Categorical baseline characteristics were compared using Fisher exact tests because of sparse cell counts, and continuous baseline summary scores were compared using independent 2-tailed *t* tests. Pre-post changes were examined using linear mixed effects models with a random intercept for participant and a fixed effect of time, baseline vs postintervention, fitted separately for each outcome [59]. This approach allowed us to use all available observations from the 109 baseline respondents and the 70 participants with postintervention data. For each model, we report the fixed effect of time as B, SE, 95% CI, and *P* value.

Because not all baseline participants completed the postintervention survey, we conducted a conservative sensitivity analysis using baseline observation carried forward (BOCF) [60]. For participants without observed follow-up data, the baseline value for each outcome was carried forward to the postintervention time point. Pre-post comparisons were then repeated in the full 109-participant sample to examine whether the overall pattern of findings was sensitive to this stringent missing-data assumption.

#### Exploratory Analyses of Perceived App Quality

To explore whether perceived app quality was associated with more favorable postintervention attitudinal outcomes, we conducted baseline-adjusted linear regression analyses using postintervention uMARS data. To provide a focused exploratory model and reduce multiple-testing burden, we used the overall mean uMARS score, calculated as the mean of the 5 uMARS subscale scores: engagement, functionality, aesthetics, information, and subjective quality, rather than entering the individual subscales separately [61].

Exploratory regressions were conducted for the 2 positive-valence outcomes most directly aligned with the platform’s intended attitudinal targets: positive Attitudes Toward Seeking Professional Psychological Help (ATSPPH_Pos) and positive expectations about counseling (Positive Expectations About Counseling Scale [BEACS_Pos]). For each model, the corresponding baseline score was entered as a covariate, and the total uMARS score was entered as the predictor of interest. We report unstandardized regression coefficients, SEs, 95% CIs, and standardized coefficients. Model diagnostics included assessment of multicollinearity and residual distributions [62].

### Integration of Qualitative and Quantitative Strands

Integration between the qualitative and quantitative strands occurred through both study design and final interpretation [63]. First, integration occurred through building, as Phase 1 findings were used by the design, development, and counseling-content teams to refine the platform’s content structure, navigation, and feature emphasis before Phase 2. Second, integration occurred at the interpretation and reporting stage, where qualitative themes were compared with quantitative patterns to examine areas of convergence and complementarity. To make this integration explicit, we provide a mapping table linking major Phase 1 themes, corresponding design revisions, and related Phase 2 evaluation domains.

## Results

### Comparison With Existing Digital Mental Health Platforms

We reviewed Fruto alongside TimelyCare [35], YOU at College [36], and IntelliCare for College Students [25] using a study-specific adaptation of the Lancet Digital Health app evaluation framework. Based on publicly available information, Fruto appeared to integrate multiple campus-linked functions within a single platform, particularly self-screening, psychoeducation, counseling access, program registration, and counseling center information. Its relative limitations were more apparent in interoperability-related functions, including data export or sharing capability. This comparison is presented to contextualize Fruto’s design positioning rather than to make formal claims of superiority over other platforms. The full comparison is provided in Multimedia Appendix 2.

### Phase 1: Qualitative Findings

#### Participant Demographics

A total of 16 students, including both undergraduate and graduate students, participated in Phase 1. Participants ranged in age from 18 to 31 years, with a mean age of 24.25 (SD 4.37) years. The majority were female (n=11, 68.8%), while 5 (31.2%) participants were male. Half of the participants were undergraduate students (n=8, 50%) and half were graduate students (n=8, 50%). Table 2 summarizes participants’ demographic characteristics and assigned vignette scenarios.

**Table 2:** Participant demographics and assigned vignette scenarios.

ID	Sex	Age (years)	Academic status	Vignette
P1	Male	18	Undergraduate	Peer supporter, proactive maintainer
P2	Female	19	Undergraduate	Help seeker, curious explorer
P3	Female	30	Graduate	Help seeker, peer supporter
P4	Female	31	Graduate	Curious explorer, help seeker
P5	Male	22	Undergraduate	Help seeker, curious explorer
P6	Female	23	Graduate	Proactive maintainer, peer supporter
P7	Male	27	Graduate	Peer supporter, curious explorer
P8	Female	28	Graduate	Help seeker, proactive maintainer
P9	Male	22	Undergraduate	Curious explorer, peer supporter
P10	Female	19	Undergraduate	Proactive maintainer, help seeker
P11	Female	23	Undergraduate	Peer supporter, curious explorer
P12	Female	29	Graduate	Proactive maintainer, help seeker
P13	Female	28	Graduate	Curious explorer, proactive maintainer
P14	Female	28	Graduate	Peer supporter, proactive maintainer
P15	Female	20	Undergraduate	Proactive maintainer, help seeker
P16	Male	21	Undergraduate	Curious explorer, peer supporter

#### Findings

##### Platform-Based Online Service Experience

###### Overview

This section explores participants’ experiences with the platform’s online features, such as mental health news and self-screening tools, as well as their perceptions of the platform’s usability and perceived impact.

###### Increased Trust Through Involvement of Campus Mental Health Professionals

Many participants reported that knowing the platform was developed in collaboration with the campus counseling center enhanced their trust in it. As 1 participant remarked, “If I hear this app was developed by the health center, I naturally trust it” (P2, HS). Participants particularly valued the impression that the mental health content was created or reviewed by professionals, such as school counselors and psychiatrists. One participant explained, “Since this app was developed by our campus health center and the articles are written by experts, I trust the content” (P3, HS).

Some users reported that this sense of trust allowed them to engage with the content with less skepticism. For example, 1 participant noted, “If the information comes from this app’s health news section, I’d trust it and read it without needing to filter it” (P4, CE). Others emphasized that citation and professional terminology enhanced the credibility of the content. As 1 participant stated, “The presence of citations in the mental health articles made them feel trustworthy” (P12, PM).

However, not all participants accepted the content uncritically. For instance, P9 expressed a more cautious perspective, stating that they would not assume everything was “100% accurate,” but still considered the content potentially helpful when supporting others. “I’m not sure if something like ‘how to overcome depression’ would really work, but if a friend needs help, I might look into it” (P9, PS).

###### Emotional Relief and Action Guidance Through Content Linked to Self-Screening Results

Participants responded positively to the way self-screening results were followed by relevant content and counseling recommendations. Several reported feeling emotionally reassured and better equipped to manage their concerns. For example, P12 noted, “After completing the self-assessment, related articles appeared. It gave me the sense that I could resolve my problems” (P12, PM). Similarly, P9 appreciated how the integrated features lowered the barrier to help-seeking: “After doing the anxiety test, I got an article about real counseling cases. It helped me understand how counseling works, and I think this kind of experience might change how hesitant friends perceive it” (P9, HS).

###### App as a Tool for Supporting Friends

Based on their experience with the app, several participants said they would recommend it to friends struggling with mental health issues. P7 stated, “A lot of my friends at school suffer from stress due to relationships or academics. I think this app would really help them” (P7, PS). P4 emphasized the app’s credibility and accessibility due to its institutional backing: “Since it’s run by the school, anyone can use it. If a friend needs help, I would say, ‘Why not try the self-assessment?’” (P4, CE).

However, some participants expressed concern that recommending a “mental health app” could carry stigma. P13 explained, “I might say it could help, but I’d worry they’d think I’m implying there’s something wrong with them” (P13, CE). P10 suggested that a more indirect approach might be more effective: “There is still a lot of stigma around mental health. So rather than saying, ‘You need help, use this app,’ I would say, ‘The school was promoting this, so I tried it out.’” (P10, HS).

##### Experience Integrating Online and Offline Services

###### Overview

This section highlights participants’ experiences with Fruto’s functions for scheduling counseling appointments and registering for offline wellness programs, including meditation sessions and group counseling.

###### Lowering Barriers to Help-Seeking

Several participants noted that the app’s bright, user-friendly interface helped reduce the psychological burden of seeking counseling. P4 explained, “I do not think my issue is that serious, so visiting the counseling center feels like a big step. But the app auto-fills my name and information, which makes it easier to give it a try.” (P4, CE).

P3 contrasted the app-based process with past experiences: “In the past, writing my information on paper at the center felt burdensome. But typing out my concerns through the app helped me organize my thoughts and reduced the fear of booking a session” (P3, HS). Some participants also suggested including brief information about what to expect during counseling, such as confidentiality policies or whether counseling records affect academic records, to help alleviate anxiety before a first visit.

###### Selective Use Based on Personal Situations

Participants assigned to the CE or PM scenarios sometimes chose not to use certain features, explaining that they did not feel an immediate need for mental health support. P5 stated, “From the perspective of a CE user, I did not need to book a session, so I did not try that feature” (P5, CE), but added they would consider using it if future issues arose. Similarly, P2 noted, “Even if I am in the CE scenario now, I might become an HS type later. Just knowing these features are available is helpful” (P2, CE), suggesting that preexposure to resources may encourage future help-seeking.

##### Design Requirements

This section identifies usability issues, technical limitations, and user recommendations for improving the platform.

###### Challenges With Text-Heavy Content and Complex Structure

Some participants appreciated the conversational tone of the health news articles, finding them easier to read. However, others felt that the amount of text was overwhelming. P9 commented, “People are used to short content such as Shorts or Reels. Long texts can feel burdensome” (P9, CE). One participant noted that the use of clinical terms made some articles difficult to understand and suggested using simpler language. Additionally, some participants found the content categorization confusing. P5 remarked, “There is so much in the health news section that it feels unorganized. You might not even notice the user reviews. It should just focus on actual health information” (P5, HS).

###### Broadening Content to Include Self-Discovery and Everyday Topics

Participants, particularly those in the CE and PM scenarios, expressed a desire for more accessible and exploratory content. P8 suggested, “Instead of only deep mental health assessments, it’d be nice to have something like an MBTI test, something for self-exploration even without serious issues” (P8, PM). Others expressed interest in topics such as self-esteem, interpersonal relationships, and learning styles. As P8 noted, “A simple learning style test would be fun. Both undergraduate and graduate students would be interested.”

Participants also expressed interest in lighter, more approachable offline programs. P15 shared, “I do not have a mental disorder, so I’d prefer relaxing activities, maybe something like a mood lamp-making session” (P15, PM). HS participants emphasized that programs focused on self-understanding should be offered individually rather than in groups. As P3 noted, “A one-on-one program to build self-esteem would be ideal” (P3, HS).

###### Minor Functional Errors

Some participants encountered functional issues, such as pages failing to load or back buttons not working as intended. These technical issues were documented and addressed during the refinement process.

#### Improvements to the Fruto App and Phase 2 Experimental Design

The issues and suggestions identified during the interviews were promptly communicated to the development team and service providers. Most UI-related improvements were subsequently implemented, including the addition of images, paragraph breaks, and bolded key points to improve readability. Additionally, the content-classification system was simplified to improve navigation.

Participants expressed a desire for content that was more relatable and focused on everyday topics such as self-regulation, self-esteem, and attention. They also preferred user-friendly language and practical, real-life examples. In response, new sections, such as “Self-Care,” were added to better address these needs.

The improved version of Fruto was launched campus-wide in September 2024. Qualitative feedback from Phase 1 directly informed app development, streamlining the design process for developers and administrators while enhancing the overall user experience. Within the first 8 weeks after launch, approximately 400 accounts were registered on the platform, providing contextual information on early campus uptake. However, the quantitative analysis was restricted to the 109 students who completed the baseline survey, of whom 70 also provided postintervention data after 8 weeks. Primary repeated-measures analyses used all available observations from these baseline and follow-up assessments.

**Figure 3:**
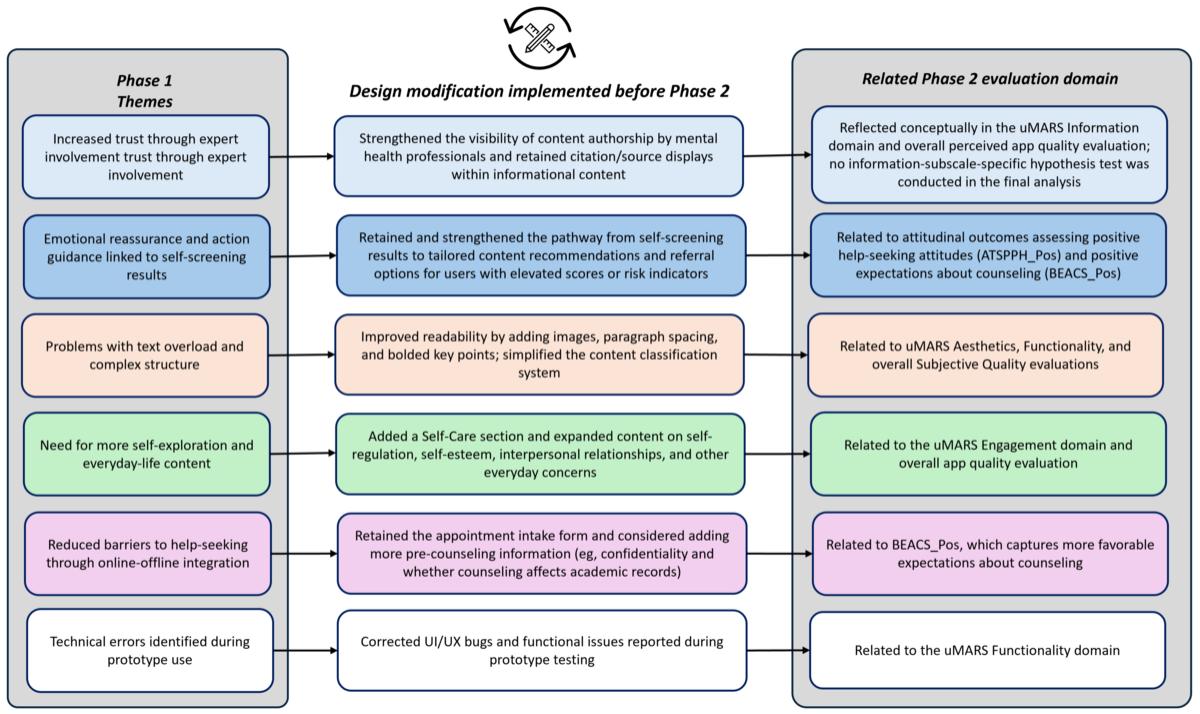
Mapping of Phase 1 qualitative themes, corresponding design refinements, and related Phase 2 evaluation domains. UI: user interface; uMARS: user version of the Mobile App Rating Scale; UX: user experience.

To clarify how Phase 1 informed subsequent platform refinement and Phase 2 evaluation, we mapped key qualitative themes onto concrete design modifications implemented before campus-wide launch and identified the corresponding domains examined in the quantitative phase. This approach is consistent with user-centered digital health research, in which user feedback, iterative testing, and implementation-focused refinement are used to improve acceptability, usability, and contextual fit before broader deployment. Figure 3 maps the Phase 1 qualitative themes, corresponding design refinements, and related Phase 2 evaluation domains.

### Phase 2: Quantitative Results

#### Background Characteristics

A total of 109 students completed the preintervention survey, and 70 provided postintervention data after 8 weeks. Because the primary repeated-measures analyses used all available observations, the quantitative analyses included baseline data from all 109 respondents and follow-up data from the 70 participants who completed the postintervention survey. The baseline sample had a mean age of 22.27 (SD 2.96) years, included 62 (56.9%) female participants, and comprised 71 (65.1%) undergraduate students. Among the 70 participants with follow-up data, the mean age was 22.34 (SD 3.13) years, 39 (55.7%) were female, and 45 (64.3%) were undergraduates. To assess potential attrition bias, we compared participants who completed only the preintervention survey (n=39) with those who also provided postintervention data (n=70). No statistically significant baseline differences were observed in sex (Fisher exact test, *P*=.84), academic status (Fisher exact test, *P*=>.99), total help-seeking attitudes (*P*=.50), or total counseling-related beliefs (*P*=.47), suggesting no clear evidence of systematic baseline differences on the measured variables. Baseline characteristics are summarized in Table 3.

**Table 3:** Baseline characteristics at week 0.

Variables			Baseline sample (n=109)		Participants with follow-up data (n=70)
**Sex, n (%)**					
	Male	47 (43.1)		31 (44.3)	
	Female	62 (56.9)		39 (55.7)	
**Age (years)**					
	Mean (SD)	22.27 (2.96)		22.34 (3.13)	
	Median (range)	22 (18-31)		22 (18-30)	
**Education level, n (%)**					
	Undergraduate	71 (65.1)		45 (64.3)	
	Graduate	38 (34.9)		25 (35.7)	

The psychometric properties of the instruments in our study were assessed by calculating Cronbach α, AVE, and CR using baseline data from the 109 participants who completed the preintervention survey. The subscales generally demonstrated acceptable reliability, supporting their use in these analyses.

BEACS_Pos showed high internal consistency (Cronbach α=0.88; CR=0.87) and adequate convergent validity (AVE=0.53). Negative Endogenous Beliefs About Counseling Scale (BEACS_Neg) and Socially Supportive Beliefs About Counseling Scale (BEACS_Social) also showed good internal consistency, with Cronbach α values of 0.81 and 0.80, respectively. BEACS_Social additionally demonstrated adequate convergent validity (AVE=0.56; CR=0.83), whereas BEACS_Neg showed adequate CR=0.82 but lower convergent validity (AVE=0.44). ATSPPH_Pos showed acceptable internal consistency (Cronbach α=0.76; CR=0.78), although its AVE was below the conventional threshold (AVE=0.42).

In contrast, negative Attitudes Toward Seeking Professional Psychological Help (ATSPPH_Neg) showed weak psychometric performance, with low internal consistency (Cronbach α=0.44), low convergent validity (AVE=0.17), and low CR (CR=0.46). Accordingly, findings involving ATSPPH_Neg should be interpreted cautiously, and the absence of a significant time effect for this subscale should be regarded as inconclusive rather than as evidence of no change. Detailed reliability and convergent validity metrics are presented in Table 4.

**Table 4:** Reliability and convergent validity of baseline outcome measures (n=109).

Construct	Cronbach α	AVE^a^	CR^b^
ATSPPH_Pos^c^	0.76	0.42	0.78
ATSPPH_Neg^d^	0.44	0.17	0.46
BEACS_Pos^e^	0.88	0.53	0.87
BEACS_Neg^f^	0.81	0.44	0.82
BEACS_Social^g^	0.80	0.56	0.83

^a^AVE: average variance extracted.

^b^CR: composite reliability.

^c^ATSPPH_Pos: positive Attitudes Toward Seeking Professional Psychological Help.

^d^ATSPPH_Neg: negative Attitudes Toward Seeking Professional Psychological Help.

^e^BEACS_Pos: Positive Expectations About Counseling Scale.

^f^BEACS_Neg: Negative Endogenous Beliefs About Counseling Scale.

^g^BEACS_Social: Socially Supportive Beliefs About Counseling Scale.

#### Quantitative Changes in Help-Seeking Variables

To examine pre-post changes in key psychological constructs, we fitted linear mixed effects models using all available observations from the 109 baseline respondents and the 70 participants with postintervention data. The fixed effect of time was significant for positive attitudes toward professional help-seeking (ATSPPH_Pos: B=0.884, SE 0.284, 95% CI 0.327-1.441; *P*=.002) and positive expectations about counseling (BEACS_Pos: B=1.585, SE 0.541, 95% CI 0.526-2.645; *P*=.003). Time effects were not significant for negative attitudes toward help-seeking (ATSPPH_Neg: B=–0.157, SE 0.255, 95% CI –0.656 to 0.343; *P*=.54), negative beliefs about counseling (BEACS_Neg: B=–0.751, SE 0.434, 95% CI –1.601 to 0.100; *P*=.08), or socially supportive beliefs about counseling (BEACS_Social: B=0.254, SE 0.338, 95% CI –0.409 to 0.916; *P*=.45). Given the weak psychometric performance of ATSPPH_Neg in this sample, the null finding for negative attitudes should be interpreted as inconclusive rather than as evidence that the intervention did not affect negative attitudes. Overall, the pre-post pattern remained consistent when all available baseline data were incorporated into the mixed effects models. Observed baseline and follow-up values and fixed effects from the linear mixed effects models are presented in Table 5.

As a highly conservative sensitivity analysis, we conducted BOCF analyses, in which baseline values were carried forward to the postintervention time point for the 39 participants without observed follow-up data. The overall pattern remained unchanged: ATSPPH_Pos remained significant (mean change 0.54, 95% CI 0.16-0.92; t_108_=2.84; *P*=.005; Cohen *d*_z_=0.27) and BEACS_Pos remained significant (mean change 1.04, 95% CI 0.32-1.76; t_108_=2.86; *P*=.005; Cohen *d*_z_=0.27), whereas ATSPPH_Neg (*P*=.43), BEACS_Neg (*P*=.08), and BEACS_Social (*P*=.25) remained nonsignificant. These results suggest that the positive findings were robust even under a stringent missing-data assumption.

**Table 5:** Observed outcome values at baseline and follow-up and fixed effects from linear mixed effects models. Baseline and postintervention means are observed descriptive statistics and are shown for transparency. Inferential tests of change are based on the linear mixed effects models using all available observations.

Measure	Baseline (n=109), mean (SD)	Postintervention (n=70), mean (SD)	Time effect, B	SE	95% CI	*P* value
ATSPPH_Pos^a^	10.62 (3.12)	11.57 (2.06)	0.884	0.284	0.327 to 1.441	.002
ATSPPH_Neg^b^	9.02 (2.46)	8.93 (2.09)	–0.157	0.255	–0.656 to 0.343	.54
BEACS_Pos^c^	25.95 (5.27)	27.50 (4.74)	1.585	0.541	0.526 to 2.645	.003
BEACS_Neg^d^	6.20 (5.06)	5.53 (3.68)	–0.751	0.434	–1.601 to 0.100	.08
BEACS_Social^e^	15.18 (3.32)	15.19 (3.68)	0.254	0.338	–0.409 to 0.916	.45

^a^ATSPPH_Pos: positive Attitudes Toward Seeking Professional Psychological Help.

^b^ATSPPH_Neg: negative Attitudes Toward Seeking Professional Psychological Help.

^c^BEACS_Pos: Positive Expectations About Counseling Scale.

^d^BEACS_Neg: Negative Endogenous Beliefs About Counseling Scale.

^e^BEACS_Social: Socially Supportive Beliefs About Counseling Scale.

#### Follow-Up Analysis: Exploratory Regression Analyses of Postintervention Outcomes

Exploratory app-quality analyses focused on ATSPPH_Pos and BEACS_Pos as the 2 positive-valence outcomes most directly aligned with the platform’s intended attitudinal targets and the primary pattern of improvement observed in the mixed effect models. Because the 5 uMARS subscales were substantially intercorrelated, we conducted a focused exploratory analysis using the overall uMARS score rather than individual subscales. Among participants with postintervention uMARS data, higher overall perceived app quality was associated with higher postintervention BEACS_Pos after adjustment for baseline BEACS_Pos (B=2.444, SE 1.066; *β*=.237, 95% CI 0.317-4.571; *P*=.03). For ATSPPH_Pos, the association with overall uMARS score was positive but did not reach statistical significance after adjustment for baseline ATSPPH_Pos (B=0.761, SE 0.434; *β*=.170, 95% CI –0.106 to 1.627; *P*=.08). These analyses were exploratory and should be interpreted cautiously.

## Discussion

### Potential of a Multidomain Platform to Lower Barriers to Help-Seeking

The main quantitative findings from Phase 2 aligned with patterns observed in Phase 1, particularly the qualitative findings presented in the “Lowering Barriers to Help-Seeking” section, where participants described reduced hesitation and lower psychological burden around seeking support. Specifically, students showed small but statistically significant improvements in positive attitudes toward professional help-seeking and positive expectations about counseling after 8 weeks of Fruto use.

In exploratory baseline-adjusted analyses, higher overall perceived app quality was associated with more favorable postintervention positive counseling expectations, while the corresponding association with positive help-seeking attitudes did not reach statistical significance. Given the exploratory nature of these analyses and the absence of objective behavioral indicators, these findings should be interpreted as preliminary self-reported changes in attitudes and counseling-related perceptions rather than evidence of behavioral change. Nevertheless, they are meaningful in light of current trends showing that students often turn to the internet for mental health information before seeking professional help [64]. Self-directed online exploration can increase accessibility, but it may also expose students to unverified, inconsistent, or low-quality content [65]. In contrast, Fruto’s professionally curated and locally tailored content may have helped lower barriers by providing credible guidance within a trusted campus service environment.

### Structural Design Principles for Multidomain Help-Seeking Platforms

The qualitative findings further suggest that the value of a multidomain platform lies not only in the number of help-seeking functions it offers, but also in how seamlessly these features are interconnected. Participants highlighted that this smooth integration, where one function naturally leads to another, enhanced their sense that support options were connected across different stages of the help-seeking process. For example, after completing a self-assessment, students were directed to tailored articles or given the option to schedule counseling appointments based on their screening results or indicated needs.

Our descriptive comparison with existing university-oriented platforms, such as TimelyCare, YOU at College, and IntelliCare for College Students, also supports this interpretation. Fruto’s potential contribution appears to lie less in any single novel component than in the integration of self-screening, psychoeducational content, counseling access, program registration, and service information within one campus-linked platform, whereas these functions are often distributed across separate university services or tools.

A recognized challenge for multidomain platforms is that integrating several functions into one system can increase cognitive burden if navigation, information structure, and transitions between features are not carefully designed [66]. In the early version of Fruto, participants identified areas where dense content organization and nested categories could make browsing feel overwhelming. Rather than indicating a limitation of the multidomain approach itself, these findings highlighted the importance of user-informed refinement to ensure that the platform remained easy to navigate. Through the Phase 1 prototype sessions, these issues were identified before campus-wide deployment and translated into design improvements, including simplified content classification, clearer feature pathways, and more context-sensitive links between self-screening, information, and counseling access. This process helped preserve the main advantage of a multidomain platform: offering multiple forms of support while maintaining a coherent and low-burden user experience [67].

### Contextual Design Principles for Everyday and Low-Stigma Use of Help-Seeking Platforms

Previous studies suggested that university students may be more willing to engage with mental health services when they are framed using positive and familiar terms, such as well-being or self-care, rather than explicitly clinical language [68]. Similar work also suggests that this framing can make digital tools feel more approachable and less stigmatizing [25]. Our qualitative findings extend this evidence in the context of a campus-integrated, multidomain platform. Although Fruto was initially designed to deliver mental health content and programs addressing common student concerns such as depression, anxiety, and sleep disturbances, participants also wanted tools for self-exploration and lighter, everyday themes.

A multidomain platform should therefore function not only as a repository of expert knowledge but also as an accessible and relatable space for everyday use. Students preferred light, engaging content that combined professional guidance with tools for self-reflection and daily life. Content offered at different levels of depth and tone may make the platform feel more approachable than purely clinical resources. Because some participants worried that recommending a “mental health app” could carry stigma, Fruto’s broader well-being and everyday self-care content, together with its connection to university services, may help position the platform as a student well-being resource rather than only a clinical tool.

### Strengths and Limitations

This study contributes to the literature on university digital mental health and the relatively underexplored area of campus-integrated, multidomain help-seeking platforms. Fruto combines self-screening, psychoeducation, counseling access, program registration, and service navigation within one system. Unlike stand-alone digital mental health tools, it was evaluated as part of a real university counseling ecosystem, allowing us to examine both user experience and preliminary changes in help-seeking attitudes and counseling-related beliefs.

The platform design was also linked to theory-informed help-seeking mechanisms, allowing each feature to be understood as targeting different but complementary pathways toward support use. The sequential mixed methods design allowed Phase 1 findings to inform concrete platform refinements before Phase 2 evaluation, making the study more closely connected to real implementation needs. In addition, the study provides actionable design guidance for universities seeking to connect digital tools with existing counseling services, including expert visibility, low-burden navigation, everyday self-care content, and clearer pathways from screening to support.

The main quantitative findings were also supported by a conservative BOCF sensitivity analysis, in which the positive outcomes remained significant even when baseline scores were carried forward for participants without follow-up data.

The vignette-based prototype sessions were also a methodological strength. By asking participants to engage with Fruto through scenarios involving seeking help for oneself, supporting a friend, exploring out of curiosity, and maintaining well-being, the interviews elicited a wider range of responses than direct questioning alone may have produced. Participants were not limited to describing only their current personal needs, but could imagine how the platform might be used across different help-seeking situations. This helped identify concrete design requirements for future multidomain platforms, including seamless integration between features, low-burden navigation, visible expert involvement, and content that supports self-understanding and preventive mental health care.

Several limitations should be noted. The quantitative component used a single-group pre-post design without a control group, which limits causal interpretation. Changes in help-seeking attitudes and counseling expectations may have been influenced by unmeasured factors, including natural adaptation to university life during the study period. All outcomes were self-reported, and no objective behavioral indicators, such as app usage intensity, feature-level engagement, counseling appointment completion, or service use, were analyzed. Accordingly, we could not determine how intensively participants used the platform or which features were most commonly used. Therefore, the results should be interpreted as preliminary changes in attitudes and counseling-related perceptions rather than evidence of behavioral change.

Sample size and attrition also require caution. Although the baseline recruitment target was met, the postintervention sample remained below the most conservative complete-case target, which may have reduced power to detect smaller effects. Because participation was voluntary, the sample may also overrepresent students already open to digital mental health tools or university-based support. In addition, ATSPPH_Neg showed weak psychometric performance, and the Korean translation of the uMARS was used for exploratory purposes only. These issues limit the interpretation of negative attitude outcomes and app-quality analyses.

Finally, Fruto was developed and evaluated in a single university context, with specific counseling-center infrastructure, institutional trust, and service workflows. Some features, such as campus-specific content and therapist-representing character mascots, may not generalize directly to other institutions or cultural contexts. Cross-campus deployment and scalability were not formally evaluated. Future studies should include control groups, objective usage and service-use data, validated app-quality measures, and more diverse institutional settings.

### Conclusion

Fruto offers a practical example of how universities can organize digital support around students’ different help-seeking needs rather than relying on isolated tools. The platform brings together self-screening, credible mental health information, self-care content, counseling access, program registration, and service navigation within one campus-integrated system. In doing so, it may help create a clearer and less burdensome pathway from early awareness to professional support.

The vignette-based interviews showed that students valued professionally developed content, personalized feedback after self-screening, and direct links to counseling and offline programs. Their feedback led to concrete refinements before campus-wide deployment, including a simpler information structure and expanded content on self-discovery and everyday concerns. After 8 weeks of use, students showed significant improvements in positive help-seeking attitudes and positive expectations about counseling, while exploratory analyses suggested that higher perceived app quality may be related to more favorable counseling expectations.

This study may encourage universities to develop connected, low-stigma digital pathways that support students before difficulties become severe and before formal counseling is needed. Future controlled studies should examine longer-term use, objective engagement, service-use outcomes, and whether improvements in attitudes and counseling expectations translate into actual help-seeking behavior.

## Appendix

Multimedia Appendix 1 Full UI screenshots and descriptions of all features.

Multimedia Appendix 2 Descriptive comparison of Fruto with existing university-oriented digital mental health platforms based on an adapted Lancet Digital Health framework.

Multimedia Appendix 3 Full interview guide.

Multimedia Appendix 4 The original vignette scripts.

## Abbreviations

ATSPPH_Neg: negative Attitudes Toward Seeking Professional Psychological Help
ATSPPH_Pos: positive Attitudes Toward Seeking Professional Psychological Help
AVE: average variance extracted
BEACS_Neg: Negative Endogenous Beliefs About Counseling Scale
BEACS_Pos: Positive Expectations About Counseling Scale
BEACS_Social: Socially Supportive Beliefs About Counseling Scale
BOCF: baseline observation carried forward
CE: curious explorer
CR: composite reliability
HS: help seeker
PM: proactive maintainer
PS: peer supporter
RQ: research question
UI: user interface
uMARS: user version of the Mobile App Rating Scale
UNIST: Ulsan National Institute of Science and Technology

## Data Availability

Data supporting the findings of this study are available upon request from the corresponding authors. The data are not publicly available due to privacy concerns.
