# Supplementary material for: Acceptability and Preliminary Evaluation of a Campus-Integrated Digital Platform (Fruto) for University Students’ Mental Health Help-Seeking: Sequential Mixed Methods Study
Source: J Med Internet Res. 2026 Jun 22;28:e78930. doi: 10.2196/78930 (PMC13338677; doi:10.2196/78930)
Supplement: Multimedia Appendix 1 [file jmir_v28i1e78930_app1.docx]

**Multimedia Appendix 1 - Full UI screenshots and descriptions of all features**

**“Fruto” App**

1. Home Screen Overview
2. Counseling Appointment System
3. Self-Screening System
4. Wellness Program Registration
5. Health News
6. Center introduction
7. **Home Screen Overview**
   - **Fruto app’s home screen interface**.


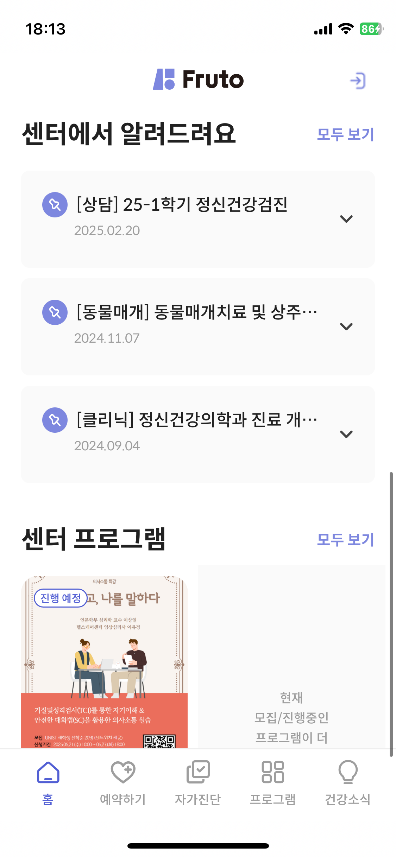

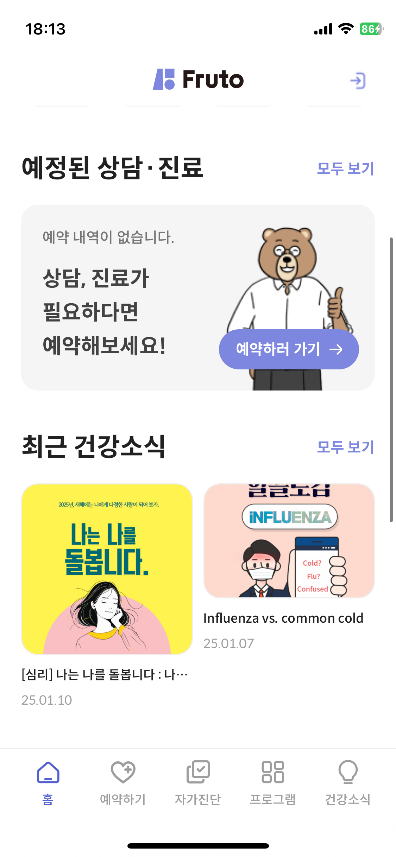

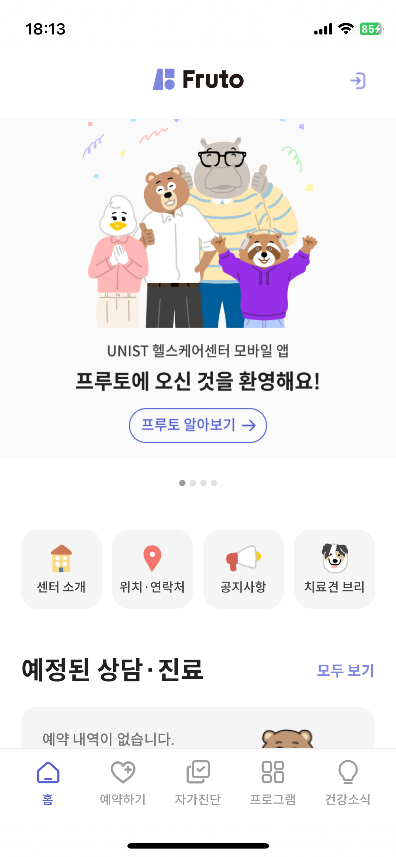


**(C)**

Announcements from the campus healthcare center and a list of ongoing wellness programs are also readily accessible.

**(B)**

Users can check upcoming counseling or clinic appointments, as well as the latest mental health news.

**(A)**

The welcome screen and navigation bar provide access to the app’s core features.

The home screen of the Fruto app features a user-friendly navigation structure, allowing easy access to the app’s core functions via a bottom tab bar. Through a scrollable interface, users can conveniently view their appointment status, mental health content, campus healthcare center announcements, and available wellness programs—all in one place.

1. **Counseling Appointment System**


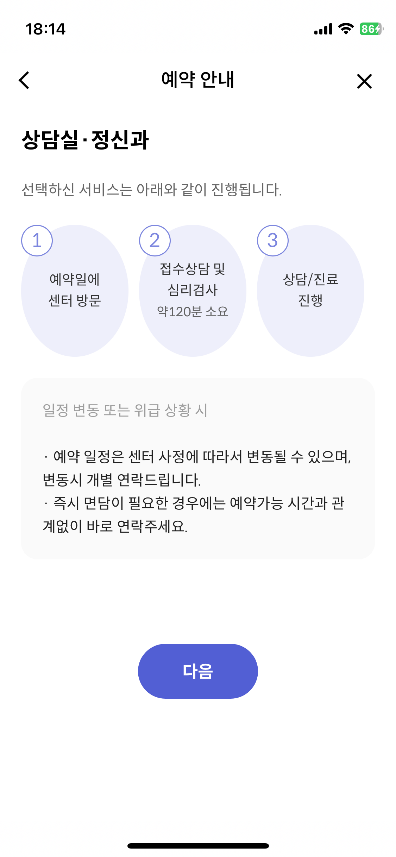
 Fruto provides a digital appointment system that allows university students to schedule psychological counseling or psychiatric consultations directly through the app, without the need for phone calls or in-person visits. This feature is designed to lower behavioral barriers to help-seeking, particularly for users who may struggle with initiating appointments due to depressive symptoms or low motivation..


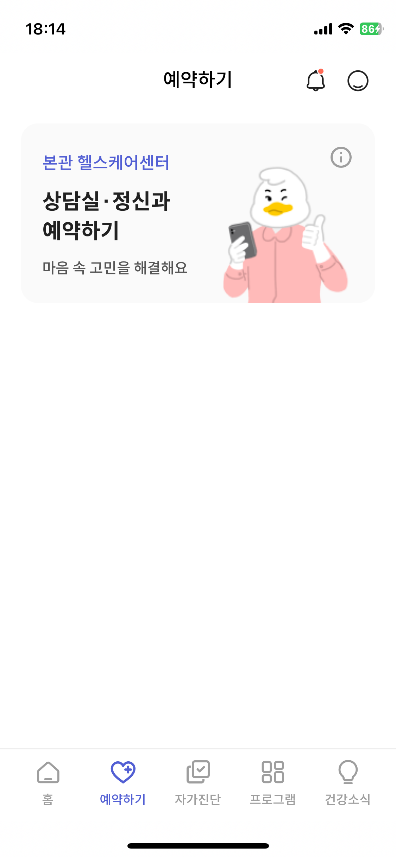

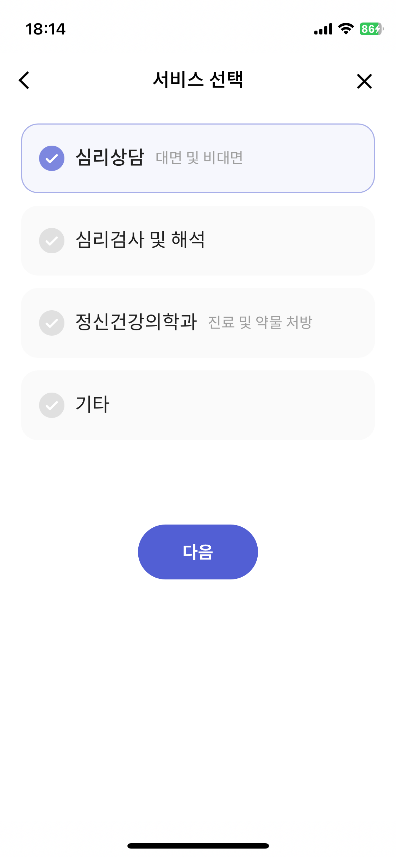


**(C)**

Instructions for proceeding with the selected service

**(B)**

Select one of the following options:

1. Psychological counseling
2. Psychological assessment and interpretation
3. Psychiatric consultation
4. Other services

**(A)**

Touch the “Appointment” tab in the navigation bar


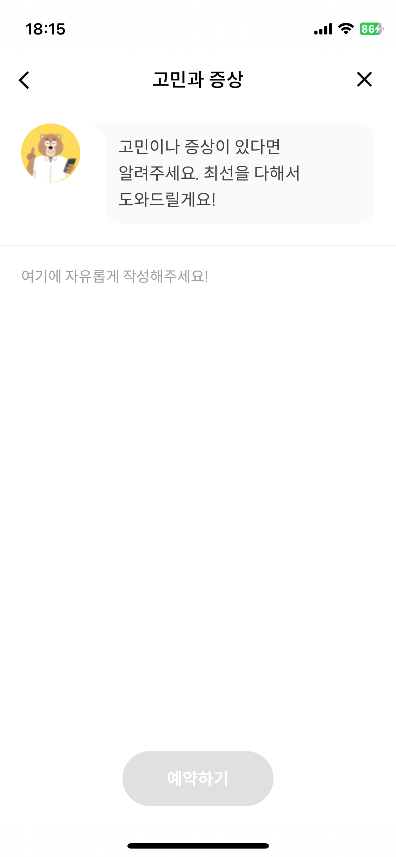

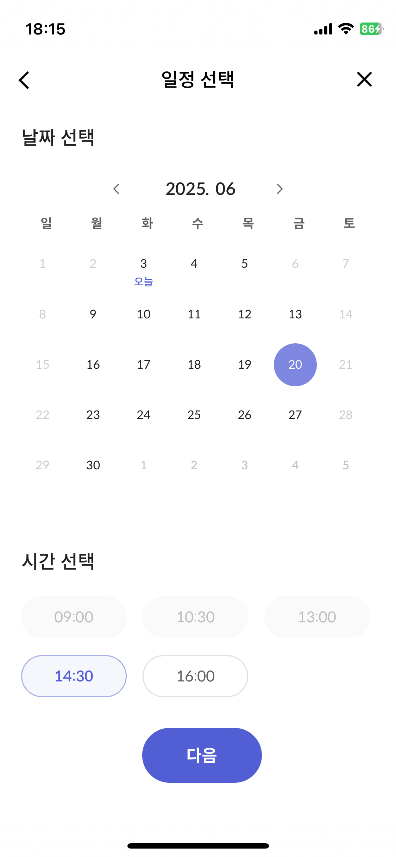


**(E)**

Free-text input of concerns and symptoms followed by appointment confirmation

**(D)**

1. Touch the desired date

2. Select an available time slot

1. **Self-Screening System**

Fruto enables users to independently complete a variety of psychological self-assessments, providing intuitively visualized results along with personalized follow-up content. This feature supports self-understanding and serves as a bridge to further counseling or self-care.
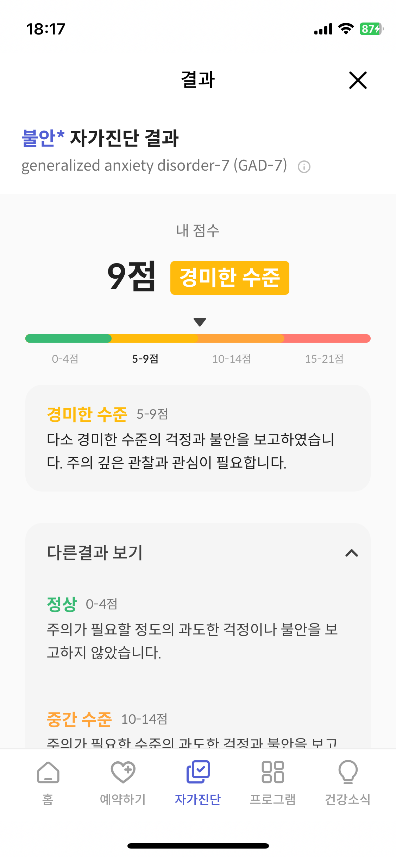

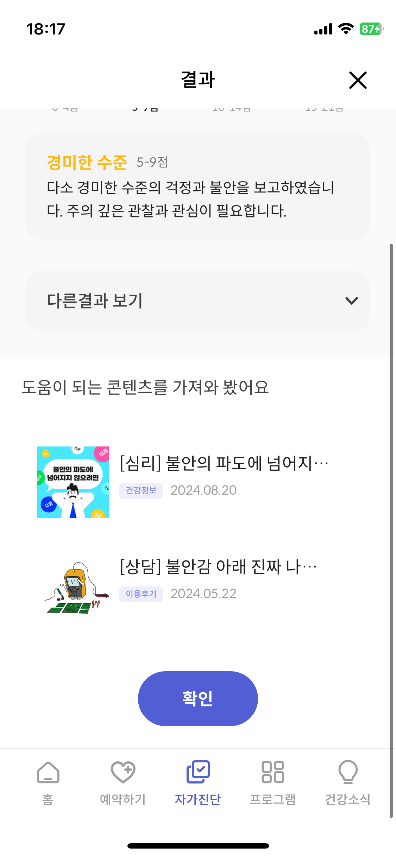

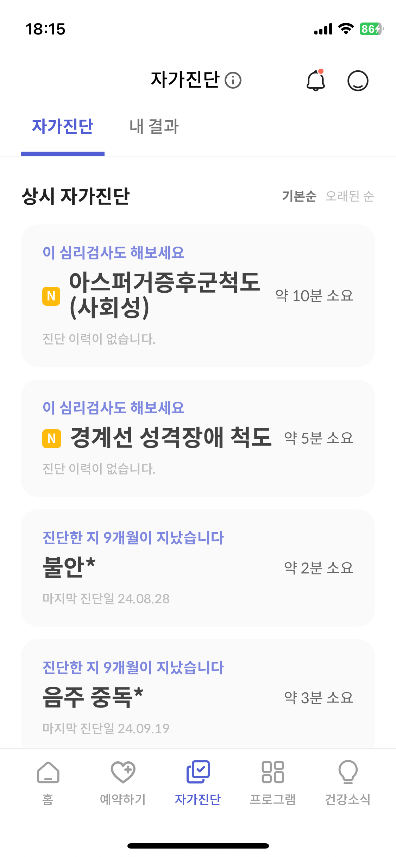


**(C)**

Personalized mental health content is automatically recommended based on the user's assessment outcomes.

**(B)**

Test outcome presentation using color-coded score ranges and level-specific interpretations

**(A)**

Touching the “Self-Assessment” tab brings up a list of available psychological tests.

1. **Wellness Program Registration**

The wellness program registration feature provides information on group counseling sessions, workshops, and special lectures offered to university students, and allows users to register directly through the app. This function is designed to complement the limitations of offline promotion, improve accessibility to programs, and encourage
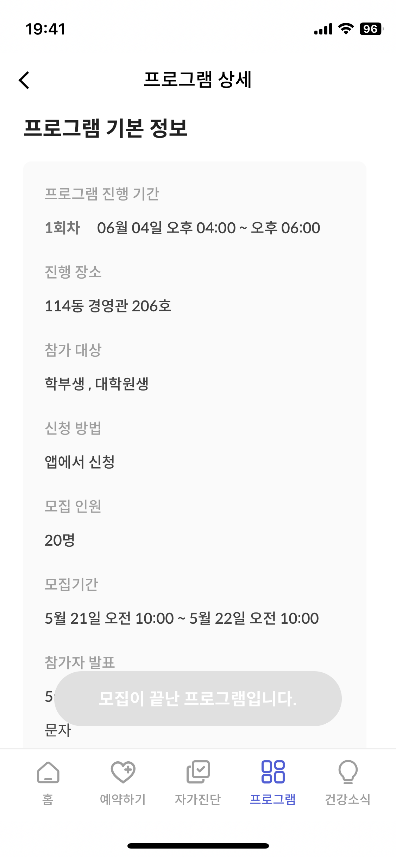

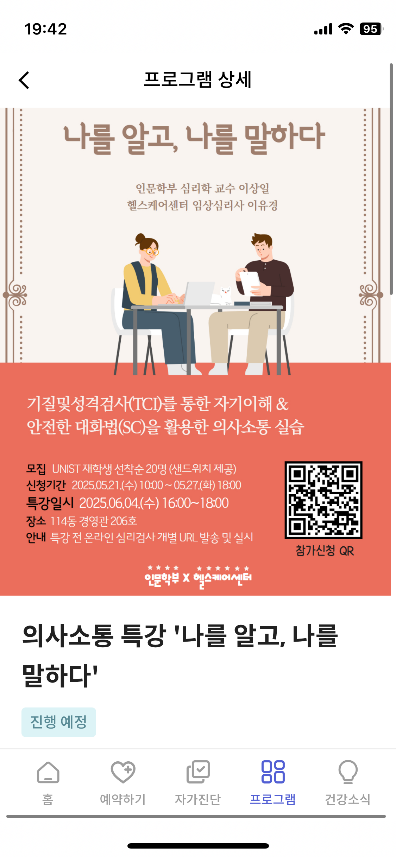

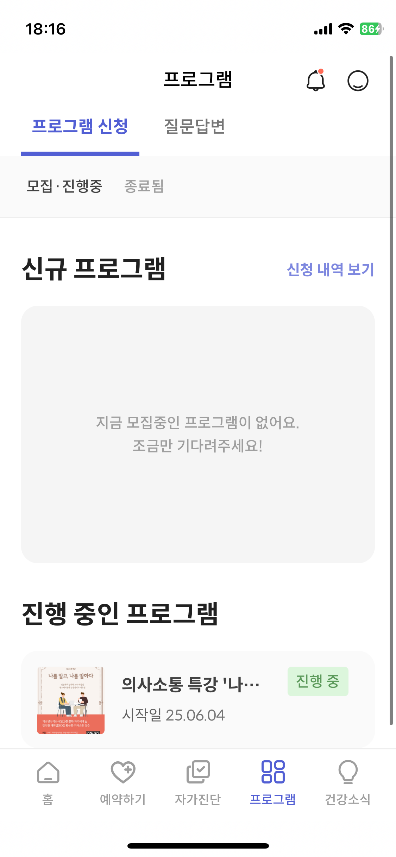
voluntary student participation.

**(C)**

Program details including schedule, location, target participants, and application period, with in-app registration via the “Apply” button

**(B)**

Access to program details and posters by selecting a program card

**(A)**

List of newly added and ongoing wellness programs via the “Programs” tab

1. **Health News**

To promote mental health literacy and strengthen students’ self-care capabilities, Fruto offers article-based content focused on mental health awareness and personal well-being.

**(A)**

내비게이션 바의 “프로그램” 탭을 터치하면 신규 프로그램과 진행 중인 프로그램 리스트를 확인할 수 있음


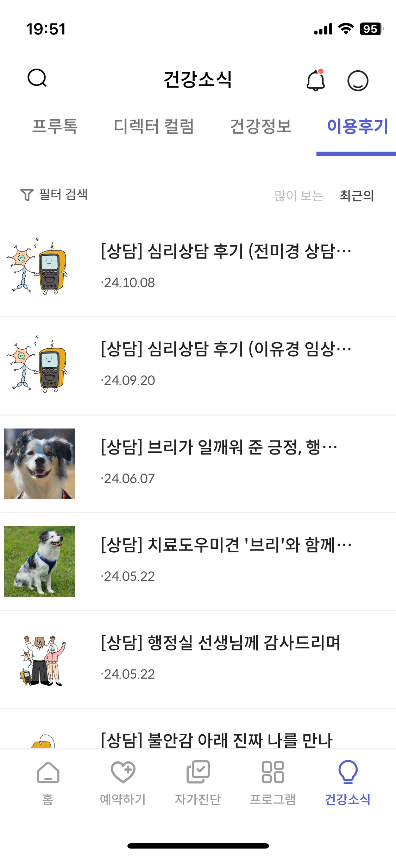

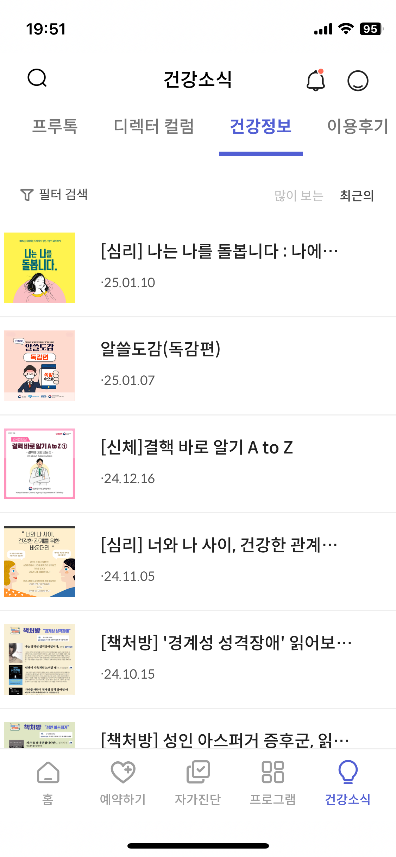

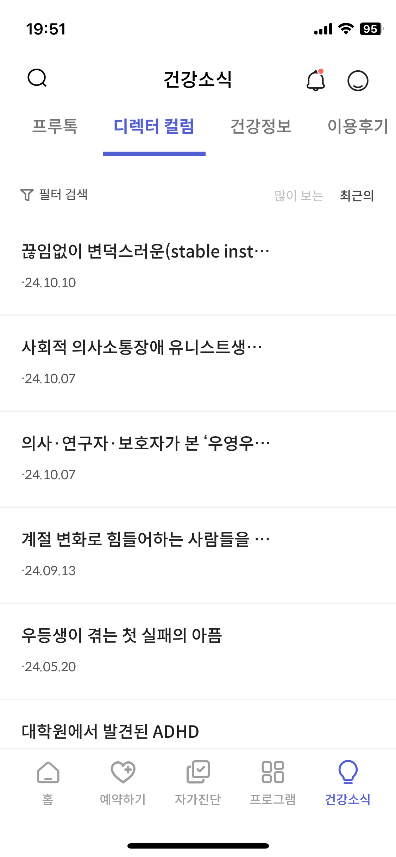

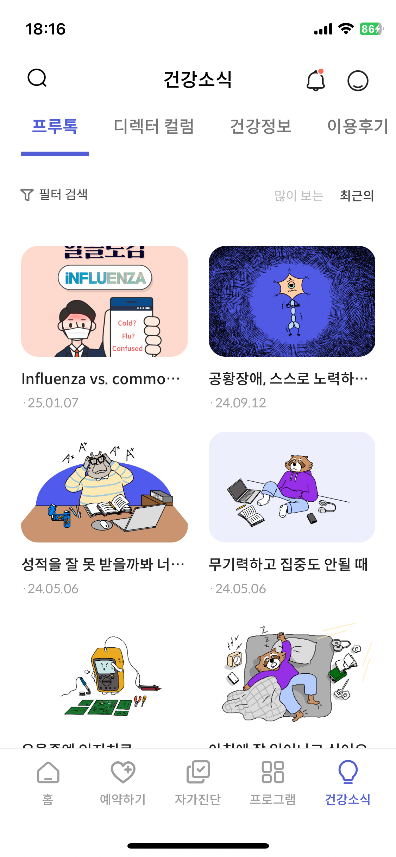


**(C)**

- **Health Information**: Resources on psychological and physical health, as well as recommended readings

**(D)**

- **User Reviews**: Shared experiences from students who have used counseling or psychiatric services

**(A)**

Access to various categories via the top navigation tabs:

- **Frutalk**: Articles written by counseling center professionals addressing common student concerns

**(B)**

- **Director’s Column**: Expert commentary from a professor of psychiatry

1. **Center Introduction**

Fruto provides a dedicated section introducing the campus healthcare center, helping users access information about university-based mental health services and encouraging trust and proactive engagement with available support resources.


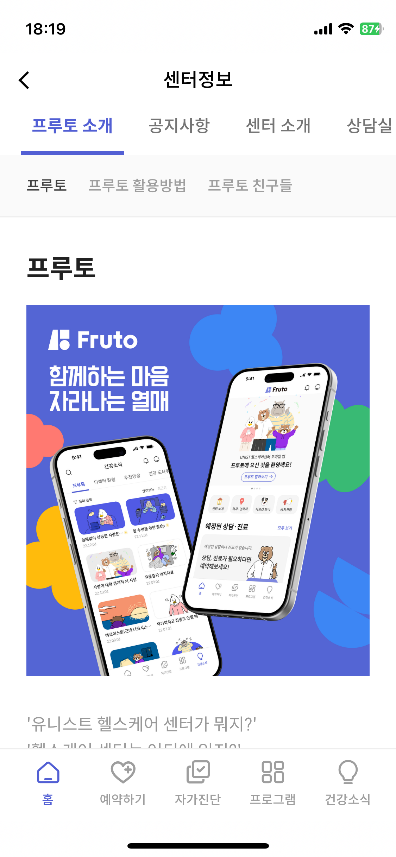

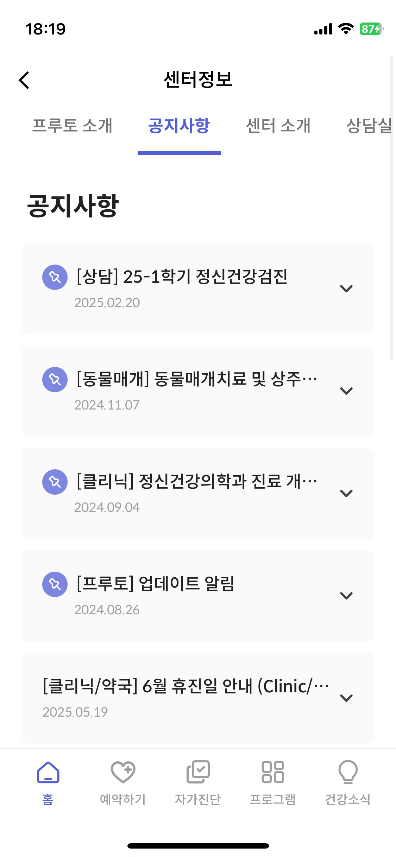

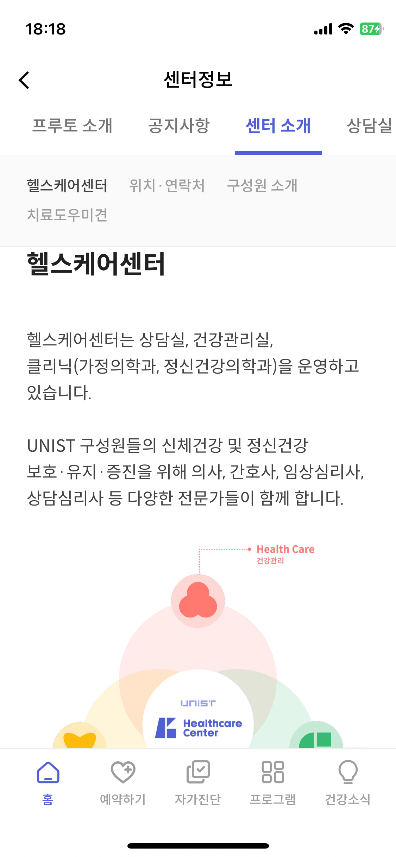


**(C)**

Introduction to How to Use Fruto

**(B)**

Latest schedules and service updates are available through the in-app announcements.

**(A)**

Campus healthcare center information: departments, location, and contact details.
