# Supplementary material for: Acceptability and Preliminary Evaluation of a Campus-Integrated Digital Platform (Fruto) for University Students’ Mental Health Help-Seeking: Sequential Mixed Methods Study
Source: J Med Internet Res. 2026 Jun 22;28:e78930. doi: 10.2196/78930 (PMC13338677; doi:10.2196/78930)
Supplement: Multimedia Appendix 2 [file jmir_v28i1e78930_app2.docx]

**Multimedia Appendix 2 - Comparison of Fruto with existing digital mental health platforms**

This table presents a comparative evaluation of three widely used digital mental health platforms for college students —intelliCare for College Students, TimelyCare, and YOU at College—based on the Lancet Digital Health evaluation framework. For contextual positioning, each criterion was coded as publicly documented, partially documented, or not identified in publicly available sources, rather than scored as a formal quality ranking. We adapted the Lancet Digital Health evaluation framework into a study-specific 3-point descriptive rubric for contextual comparison. The original framework does not prescribe summary scoring or ranking.

Publicly available materials were reviewed between April 4, 2026 and April 20, 2026 by 2 members of the research team. Initial ratings were discussed until consensus was reached. Because the comparison relied on public information, unpublished or recently updated internal features may not have been captured.

Table 1. Comparison of Fruto with existing digital mental health platforms based on the Lancet Digital Health framework

|  | Applications | | | |
| --- | --- | --- | --- | --- |
| Item | Fruto | IntelliCare for College Students [a, b] | TimelyCare [c] | YOU at College [d] |
| Level 1: Privacy and Security | | | | |
| 1. Types of personal data clearly specified | 3 | 2 | 3 | 3 |
| 2. Data securely encrypted and stored | 2 | 1 | 1 | 3 |
| 3. No third-party data sharing or selling | 2 | 3 | 1 | 2 |
| 4. Privacy policy easily accessible | 3 | 1 | 3 | 3 |
| Level 2: Evidence Base | | | | |
| 1. Supported by peer-reviewed research | 2 | 3 | 1 | 2 |
| 2. Designed based on clinical guidelines | 3 | 3 | 3 | 3 |
| 3. Information sources and authors clearly stated | 3 | 3 | 3 | 2 |
| Level 3: Usability | | | | |
| 1. Usable without prior training | 3 | 3 | 3 | 2 |
| 2. Meets accessibility standards (e.g., font size, contrast) | 3 | 2 | 2 | 2 |
| 3. Functional stability (no major crashes/errors) | 2 | 2 | 2 | 2 |
| 4. Help or guidance available | 3 | 3 | 3 | 2 |
| Level 4: Interoperability | | | | |
| 1. Integration with healthcare systems or external apps | 3 | 3 | 3 | 1 |
| 2. Data export or sharing capability | 1 | 2 | 3 | 2 |
| Level 5: Engagement and Customization | | | | |
| 1. Personalized content based on user needs | 3 | 3 | 1 | 3 |
| 2. Engagement features (notifications, reminders) | 3 | 3 | 1 | 3 |
| 3. Supports diverse student populations | 3 | 3 | 3 | 3 |

1 = no supporting public evidence identified; 2 = criterion partially addressed or mentioned without sufficient implementation detail; 3 = criterion explicitly documented in publicly available materials.

References

[a] Lattie, E., Cohen, K., Winquist, N., & Mohr, D. (2020). Examining an App-Based Mental Health Self-Care Program, IntelliCare for College Students: Single-Arm Pilot Study. *JMIR Mental Health*, 7. <https://doi.org/10.2196/21075>.

[b] Lattie, E., Cohen, K., Hersch, E., Williams, K., Kruzan, K., MacIver, C., Hermes, J., Maddi, K., Kwasny, M., & Mohr, D. (2021). Uptake and effectiveness of a self-guided mobile app platform for college student mental health. *Internet Interventions*, 27. https://doi.org/10.1016/j.invent.2021.100493.

[c] TimelyCare. <https://timelycare.com/medical/>

[d] YOU at College. https://youatcollege.com/you-student-impact/
