# Supplementary material for: Acceptability and Preliminary Evaluation of a Campus-Integrated Digital Platform (Fruto) for University Students’ Mental Health Help-Seeking: Sequential Mixed Methods Study
Source: J Med Internet Res. 2026 Jun 22;28:e78930. doi: 10.2196/78930 (PMC13338677; doi:10.2196/78930)
Supplement: Multimedia Appendix 3 [file jmir_v28i1e78930_app3.docx]

**Multimedia Appendix 3-Full interview guide**

**1. Feature-specific Usage Experience and Feedback**

(*If the participant has not used the feature, explain it briefly and ask them to try it before proceeding.*)

**1.1. Counseling and Psychiatric Appointment System**

**Usage**: ☐ Yes ☐ No

If used:

- How was your experience using this feature?
- How easy was the appointment process?
- Is there anything you think could be improved in the reservation and management process?

If not used:

- What was the reason for not using this feature?
- Were you aware of this feature?
- (Based on your vignette scenario) Do you think you would use it in the future? In what kind of situation?

**1.2. Program Information and Registration**

**Usage**: ☐ Yes ☐ No

If used:

- Which program(s) did you apply for?
- How easy was the program application process?
- Was the information provided about the program sufficient?

If not used:

- What was the reason for not using this feature?
- Were you aware of this feature?
- What kind of programs would interest you if offered?

**1.3. Self-Assessment Tools**

**Usage**: ☐ Yes ☐ No

If used:

- Was the self-assessment result useful for you?
- Was the interpretation of the results easy to understand?
- Do you think the self-assessment items were sufficient?

If not used:

- What was the reason for not using this feature?
- Were you aware of this feature?
- Do you think self-assessment features like this could be helpful?

**1.4. Health News Section**

**Usage**: ☐ Yes ☐ No

If used:

- Was the information in the Health News section useful?
- What did you think of the credibility of the content?
- What kinds of health topics would you like to see more of?

If not used:

- What was the reason for not using this feature?
- Were you aware of this feature?
- What kind of health-related content would draw your interest?

**1.5. Center Introduction Section**

**Usage**: ☐ Yes ☐ No

If used:

- Was the information about the center sufficient?
- Were the location, staff, and contact details helpful?
- Was there any additional information you wished to see?

If not used:

- What was the reason for not using this feature?
- Were you aware of this feature?
- Do you think having access to center-related information is important?

**2. Overall App Experience**

(*For Q1 and Q2, ask in the context of each participant’s assigned vignette.*)

1. Overall, how was your experience using the app?
2. Which feature did you find the most helpful?
3. What aspect(s) of the app or the center do you think need improvement?
4. Would you recommend this app to a friend or peer? If yes, why?
     - What kind of person or in what situation would you recommend it?
