# Supplementary material for: Acceptability and Preliminary Evaluation of a Campus-Integrated Digital Platform (Fruto) for University Students’ Mental Health Help-Seeking: Sequential Mixed Methods Study
Source: J Med Internet Res. 2026 Jun 22;28:e78930. doi: 10.2196/78930 (PMC13338677; doi:10.2196/78930)
Supplement: Multimedia Appendix 4 [file jmir_v28i1e78930_app4.docx]

**Multimedia Appendix 4 - The original vignette scripts**

Help Seeker

During high school, you consistently ranked among the top students in your class, receiving frequent praise from teachers and friends alike. However, upon entering college, you experienced a significant setback when you failed to achieve the grades you had anticipated for the first time. Despite investing a similar amount of time and effort as your peers, the gap in academic performance weighed heavily on you. This sense of helplessness—feeling that hard work might not pay off—led to growing doubts about your career path and sense of purpose, intensifying your anxiety. In turn, these worries began to affect your daily life, creating a vicious cycle of poor concentration and further stress. While searching for a solution, you came across a campus mental health support app, and, in the hope that it might improve your circumstances, you decided to download it.

Peer Supporter

Recently, your close friend A has seemed depressed and has been unresponsive to calls and messages. You finally managed to have a brief conversation with them, and learned that since choosing their major, A has been under intense stress and feeling powerless about their academics and future plans. You want to offer direct help, but each time you try, A compares themselves to you and other friends, revealing their vulnerability. Worried that A may need professional intervention, you hear about the release of a new mental health app on campus. You decide to download the app first, explore its various features, and then recommend the ones you believe could benefit your friend.

Curious Explorer

You’ve heard news about a new mental health support app being released on campus. Although you don’t have any particular mental health concerns, you are curious about new technology and on-campus services. By trying out the app in advance, you might be able to recommend it to younger students or friends when they’re struggling. Additionally, you’re interested in receiving rewards offered to early users. So, you decide to download the app and explore its various features.

Proactive Maintainer

Although you haven’t experienced any mental health issues yourself, you once witnessed someone close to you suffer severely from depression, requiring hospital visits and medication. This experience prompted you to research various mental health conditions—including depression—and become more proactive about monitoring your own mental well-being. While looking into different apps and services that offer mental health information, you came across a new mental health app provided by your school. As a preventive measure, you decide to download the app and give it a try.
